# Load Effect of Automated Truck Platooning on Highway Bridges and Loading Strategy

**DOI:** 10.3390/s22207704

**Published:** 2022-10-11

**Authors:** Tianyang Ling, Lu Deng, Wei He, Haibing Wu, Jiayu Deng

**Affiliations:** 1 College of Civil Engineering, Hunan University, Changsha 410082, China; 2 Key Laboratory for Damage Diagnosis of Engineering Structures of Hunan Province, Changsha 410082, China

**Keywords:** automated truck platooning, vehicle load effect, highway bridge, truck loading strategy

## Abstract

Automated truck platooning (ATP) has gained growing attention due to its advantage in reducing fuel consumption and carbon emissions. However, it poses serious challenges to highway bridges due to the load effect of multiple closely spaced heavy-duty trucks on the bridge. In China, ATP also has great application prospects in the massive and ever-increasing highway freight market. Therefore, the load effects of ATP on bridges need to be thoroughly investigated. In this study, typical Chinese highway bridges and trucks were adopted. ATP load models were designed according to the current Chinese road traffic regulations. The load effects of ATP on highway bridges were calculated using the influence line method and evaluated based on the Chinese bridge design specifications. Results show that the load effect of ATP on bridges increases with the increase in the gross vehicle mass and the truck platooning size but decreases with the increasing inter-truck spacing and the critical wheelbase. The Grade-I (best quality standard) highway bridges are generally capable of withstanding the ATP loads, while caution should be exercised for other bridges. Strategies for preventing serious adverse impacts of ATP load on highway bridges are proposed.

## 1. Introduction

Highway transportation undertakes the vast majority of inland freight transport tasks in the world and accounts for nearly 75% of inland freight transport in China [1] and over 60% in many other countries. At the same time, road vehicles, especially heavy-duty trucks, are one of the primary sources of carbon emissions. According to the data [2] reported by the International Energy Agency (IEA) in 2017, the CO_2_ emissions produced by vehicles account for about 17% of global CO_2_ emissions. Therefore, in the last few years, many countries have developed laws and technical guidelines for traffic management departments in order to reduce CO_2_ emissions. For example, in China’s “13th Five-Year Plan for the Development of Modern Integrated Transportation System”, the intelligent transportation technique was determined to be a prior development direction, based on which the goal of reducing carbon emissions in transportation by 7% was made. This goal extends to the 14th Five-Year Plan.

**Figure 1:**
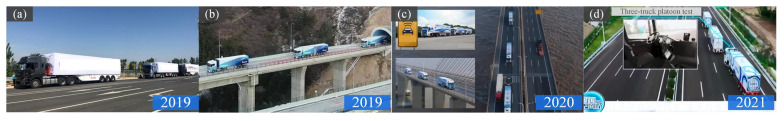
ATP tests conducted in China: (**a**) ATP public test for standard setting in Tianjin; (**b**) ATP tests on Beijing–Liquan highway; (**c**) ATP tests on Donghai Bridge in Shanghai; (**d**) ATP tests on a smart highway in Jiangsu.

Automated truck platooning (ATP) is a new intelligent and green transportation technology [3]. The technology enables multiple trucks spaced at a close distance to optimize their aerodynamic characteristics, thus reducing fuel consumption [4] and carbon emissions [5]. Promoted by the vehicle-to-vehicle (V2V) [6] and the 5G vehicle-to-everything (5G-V2X) technologies [7], the following gaps between trucks can be further shortened and maintained to reduce the aerodynamic drag. In addition, ATP can also help improve road safety, save operating costs [4], reduce congestion [5], and indirectly solve many other traffic problems [8]. For these reasons, ATP has attracted increasing attention, and its commercial applications were recently tested in many countries [9,10], especially in China, as shown in Figure 1.

Although ATP has been proven to be energy-efficient, it may induce negative impacts on existing highway bridges. An ATP formation consists of multiple closely spaced heavy-duty trucks. This can lead to the presence of multiple heavy-duty trucks on the bridge at the same time, which is significantly different from the conventional traffic loads. Specifically, according to the traffic survey data in [11], only about 2% of the following distances between vehicles in the same lane are less than 30 m. Even for two-lane bridges, the probability of multiple presence is still less than 6.7% [12], which may be much lower if only heavy-duty trucks are counted. Actually, the mandatory safe distances for ordinary vehicles are generally no less than 50 m. For heavy-duty trucks, the requirement is usually longer than 100 m [13]. Promoted by the advanced 5G, V2X communication technologies [14], and autonomous driving technology [15], the following gap between vehicles can be shortened to less than 15 m [16]. Consequently, the transportation density will be significantly increased. However, such densely platooned heavy truck loads were not considered in the bridge design methods of many countries [17], which raises an increasing public concern that the highway bridges in service may no longer be safe under such ATP loads.

This study focuses on investigating the responses of highway bridges under the action of ATP loads. Bridge models designed based on the Chinese bridge design code were adopted for a numerical study, and a comprehensive analysis of the load impact of ATP on highway bridges was performed. Typical ATP load models were developed according to the current legal limits of vehicles in China. The load effects of ATP on bridges were compared with those calculated based on the design lane loads to investigate the critical loading conditions. The effects of truck platooning size, inter-truck spacing, and truck capacity utilization rate were studied. Based on the results, a general loading strategy for ATP was proposed; this strategy can serve as a guide to minimize the adverse effect of ATP loads on bridges during operation.

## 2. Literature Review

The capacity of existing highway bridges to bear the ATP load directly affects the sustainable development of highway infrastructures and the future implementation of the ATP technology [17,18]. Recently, assessments of the impact of ATP on highway bridges have been conducted by some researchers. The Florida Department of Transportation (FDOT) [19] found that the highway bridges in Florida cannot sustain a load of a two-truck platoon in some cases. In order to evaluate the most unfavorable load effect caused by a two-truck platoon, Iatsko et al. [13] considered scenarios of a two-truck platoon with a minimum (axle-to-axle) space of 4.3 m, based on which a new live load was suggested for improving the design load provision of the existing U.S. bridge design specification. Kamranian et al. [20] further considered the impact of ATP composed of more than two trucks and conducted a field study on the Hay River Bridge in the United States. They found that the bridge only showed an adequate capacity for two-truck ATPs, while ATPs consisting of three or four trucks would lead to a load rating that exceeds the allowed limit of the bridge. In addition to the number of platooning trucks, inter-truck spacing is another important parameter that affects the ATP-induced load effect. Yarnold et al. [21,22] carried out a parametric analysis on ATP loads and considered bridges with different span configurations. The results revealed that the bridge’s controlling internal forces (including bending moment and shear force) under the ATP load can be significant, and this may restrict the implementation of ATP in the future. A report from the Texas Department of Transportation [23] also pointed out that highway bridges might require greater load-carrying capacities if ATPs with small inter-truck spacings were allowed to pass. To avoid the additional cost for bridge load-carrying capacity enhancement, Yang et al. [16] studied the minimum safe headways of ATP load for highway bridges shorter than 60 m [24]. Based on the truck platoon load models developed by utilizing a SU4 truck, Sayed et al. [25] found that the bridge internal forces caused by ATP loads can be more significant compared to those under a single-truck load. For steel bridges, Couto Braguim et al. [26] found that truck platoons with different inter-truck spacings can cause various degrees of fatigue damage to bridges, and taking proper control of the inter-truck spacing can help reduce the fatigue damage.

In summary, the ATP technique is greatly transportation-efficient and economic, but it also raises urgent concerns about the safety of existing infrastructures. However, previous research was more focused on specific traffic scenarios under the U.S. traffic regulations; in particular, the truck load models were only designed under the federal gross weight limit (80,000 lbs) [27], which is relatively low and is therefore not applicable to many other traffic scenarios [12,28]. In addition, very few factors affecting the controlling internal forces of the bridges have been considered. China has an enormous scale of highway bridges in the world and is actively promoting ATP applications under mounting pressure to reduce carbon emissions, while the truck weight regulations are not executed strictly in China. Although studies regarding ATP algorithms [6], operating control [29], and application scenarios [30,31] have been carried out in China, the ATP-induced adverse impacts on bridge infrastructures have rarely been studied.

## 3. Vehicle Loads on Highway Bridges

### 3.1. Traffic Load Models of Chinese Bridge Design Codes

#### 3.1.1. Bridge Design Code (1985)

**Figure 2:**
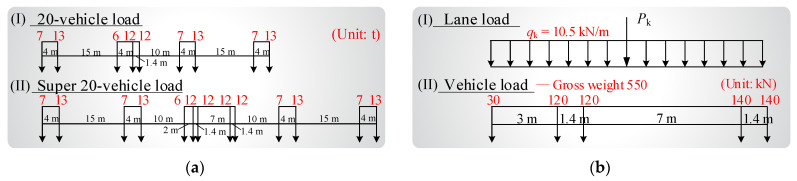
Traffic load models in the Chinese bridge design codes: (**a**) MOT (1985); (**b**) MOT (2004 and 2015).

The large-scale construction of highway bridges in China started in the 1980s. The national bridge design and construction specification adopted back then was the “general specifications for design of highway bridges and culverts (JTJ 021—85)”, in which the “20-vehicle load” and the “super 20-vehicle load” were defined as the design vehicle load models for highway bridges, as shown in Figure 2a. These traffic load models had already considered vehicle platooning scenarios, but they can only represent the mixed traffic flow composed of different vehicles (including 2-, 3-, and 5-axle vehicles) spaced at 10 or 15 m. Such load models may not be able to fully characterize the load distribution of platooning heavy-duty trucks.

#### 3.1.2. Bridge Design Code (2004 and 2015)

The traffic load models in the Chinese bridge design code were modified in 2004 (MOT 2004) and 2015 (MOT 2015) [32]. Since 2004, the standard vehicle load has been divided into two levels: Grade-I and Grade-II. Both grades of standard load models consist of two parts: the lane load and the vehicle load, as shown in Figure 2b. They are used for global structural design and local component checking purposes, respectively. The lane load consists of a uniformly distributed load (*q*_k_) and a concentrated load (*P*_k_). For Grade-I highway bridges, the standard value of *q*_k_ is 10.5 kN/m, and the standard value of *P*_k_ is determined according to Equation (1). As for Grade-II highway bridges, all the standard values should be scaled down to 75% of those of Grade-I. The vehicle load (see Figure 2(bII)) represents a five-axle vehicle with a gross weight of 550 kN.

(1) $$P_{K}=\left\{\begin{array}{l} \;180\;\;{\;}^{@2004}\\ \;4L+160\;\\ \;360 \end{array}\right.\left|\begin{array}{l} \;270\;{\;}^{@2015}\\ \;2L+260\\ \;360 \end{array}\right.\;\left|\begin{array}{l} \;\left(L\leq 5\;m\right)\\ \;(5\;m<L<50\;m)\\ \;(L\geq 50\;m) \end{array}\right.$$

in which *P*_k_ is the standard value of the concentrated force and *L* is the bridge span length.

### 3.2. Automated Truck Platooning Load

#### 3.2.1. Typical Semi-Trailer Trucks

**Figure 3:**
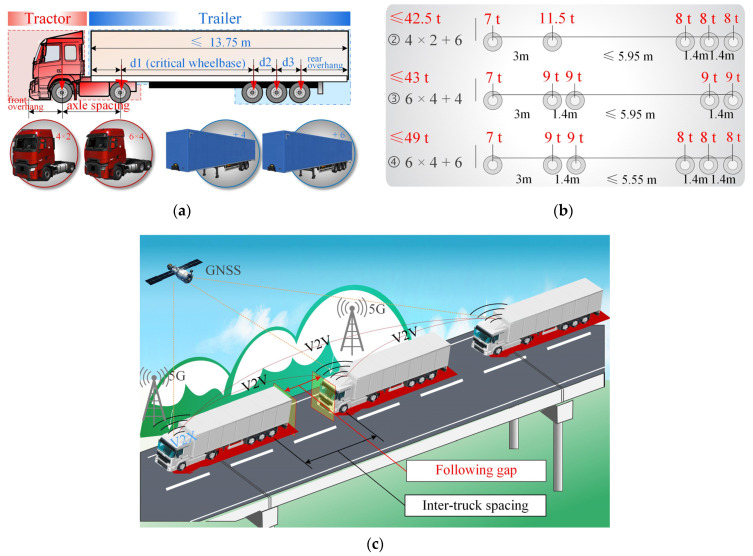
Truck platoon loads: (**a**) components of a semi-trailer truck; (**b**) dimensions, axle loads, and masses of three typical semi-trailer trucks; (**c**) schematic of an ATP crossing a highway bridge.

The semi-trailer truck (corresponding to the Class 9/10 vehicle in the United States) plays a dominant role in road freight transportation due to its good braking and manipulation performance [33]. This type of truck is also the most commonly used vehicle in ATP tests worldwide. As can be seen from Figure 3a, a semi-trailer truck generally consists of a tractor and a trailer. Apparently, the wheelbase d1 is a critical factor reflecting the load distribution of the truck, and it is referenced as the critical wheelbase hereafter.

According to the current legal limits (GB 1589—2016) [34] on masses and dimensions of a freight truck in China, three typical semi-trailer truck load models were developed, i.e., “4 × 2 + 6”, “6 × 4 + 4”, and “6 × 4 + 6”, as presented in Figure 3b. Here, “4 × 2” means that the tractor has four wheels, two of which are driving wheels; “+6” means that the trailer has six wheels (i.e., three axles). Based on the new regulations associated with overload vehicles, the upper limit of the gross truck mass for these three semi-trailer trucks is adopted as 42.5 t, 43 t, and 49 t, respectively. The upper limits on the total length, the front fitting radius, and the rear overhang of the semi-trailer are 13.75 m, 2.04 m, and 3.50 m, respectively. The axle spacing of the tractor and the axle-group spacing are assumed to be fixed at 3.00 m and 1.40 m, respectively. Then, the length ranges of the wheelbase were determined, as shown in Figure 3b.

#### 3.2.2. Platooning Truck Arrangement

Figure 3c shows the scenario of ATP crossing a highway bridge. As illustrated in Figure 3c, the vehicle speed and following gaps of the ATP can be adjusted in real time through the V2V communication system. With the help of the 5G-V2X and the Global Navigation Satellite System (GNSS), the management of the ATP can be more automotive and intelligent to accommodate the traffic conditions ahead and avoid any upcoming road accidents. For the purpose of convenience, the term “inter-truck spacing”, as illustrated in Figure 3c, is used in this study to characterize the axle-to-axle (rear to front) load spacing of the ATP. This parameter, together with the platooning size, the axle loads, and the masses of an individual truck, is recognized as a critical factor that determines the overall load distribution of the ATP.

## 4. Effects of ATP Load on Bridges

### 4.1. Assessment Method

**Figure 4:**
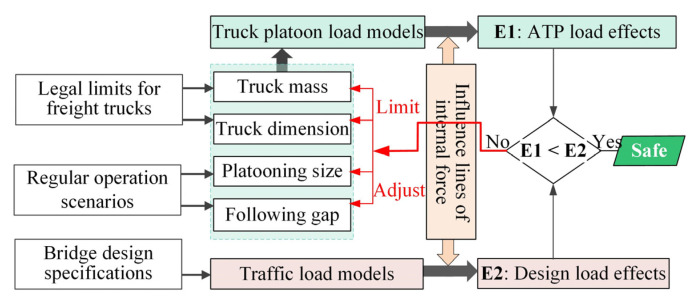
Assessment method of ATP-induced load effects.

Figure 4 provides a flow chart of the assessment method adopted in this study. Based on the standard design load models and the ATP load models developed in Section 3, the corresponding load effects can be calculated using the influence lines of the bridge. By comparing the load effects due to the ATP loads with those due to the design vehicle (lane) loads [32], the loading conditions representing bridge safety margin can be identified, just as adopted by other studies [21,22]. These conditions can serve as essential references for rational traffic decision-making, including decisions on how to manage the platooning size and gaps and to what extent the masses and dimensions of trucks should be limited.

### 4.2. Construction of Bridge Influence Lines

**Figure 5:**
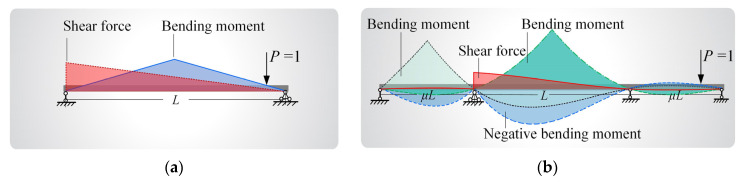
Influence lines for internal forces of (**a**) a simply supported bridge and (**b**) a continuous bridge.

Small- and medium-span simply supported bridges and continuous bridges, which are sensitive to vehicle loads [35,36], were considered in this study. The influence lines of the bridges, which represent the relationship between the internal forces and the external loads applied to the bridges, were constructed based on the analytical expressions of the controlling internal forces, as shown in Figure 5**.** Specifically, for unequal-span continuous bridges (with a main span length of *L* and a side-to-main span ratio of *μ*), the analytical expressions of the influence lines were deduced for efficient evaluation. The accuracy of the deduction results was validated using the SM Slover software. As a case illustration, Figure 5b shows the influence lines of a three-span continuous bridge with a main span of 40 m and a side-to-main span ratio of 0.6.

### 4.3. Assessment Framework

Table 1 lists the parameters and their ranges considered in this study. Three typical semi-trailer trucks as well as the design truck (55 t) were considered. To be conservative, a reduction of 0–2 m in the critical wheelbase was taken into consideration for each truck, since the critical wheelbase can be as short as 4.3 m [24]. The truck capacity utilization rate (i.e., the ratio of the true gross mass to the upper limit of mass for truck) was considered to be 60–100%. According to the automation standards [16] and ATP application experiences [13], 20 m and 4 m were defined as the standard and extremum inter-truck spacing for the ATP, respectively. The former represents a mean following gap in operation, while the latter represents an extreme following gap that may possibly occur. For continuous bridges, the side-to-main span ratio of 0.5 to 1.0 was adopted. Two types of controlling internal forces were considered for simply supported bridges, and four types of controlling internal forces were analyzed for continuous bridges, as listed in Table 1. Based on these parameters, an exhaustive numerical calculation was conducted using MATLAB software, and the responses of highway bridges under all probable ATP load scenarios were obtained.

## 5. Results and Discussion

### 5.1. Vehicle Load Effects Calculated Based on the Chinese Bridge Design Specifications

**Figure 6:**
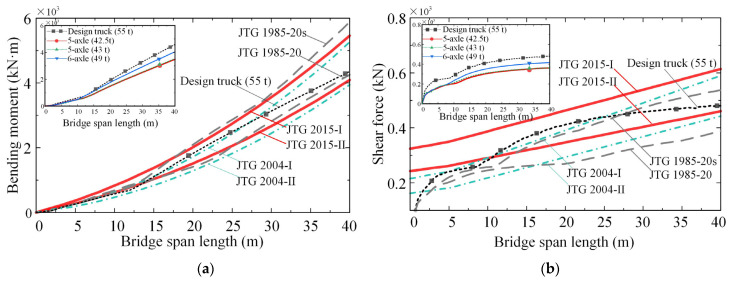
Load effects of simply supported bridges due to design vehicle loads: (**a**) bending moment; (**b**) shear force.

The maximum controlling internal forces calculated for the simply supported bridges are plotted in Figure 6. The main figures show the load effects due to the design vehicle loads in different bridge design specifications. The subgraphs plot the load effects due to a single-truck load, including the current design truck load (55 t). In both of the main figures and the subgraphs, the dashed lines with square markers represent the load effect due to the current design truck load (2015 version).

As can be seen from Figure 6, both the bending moments and shear forces calculated based on the 2015 bridge design specification are greater than those calculated with the 2004 bridge design specification, while they are close to those calculated based on the 1985 bridge specification. Similar findings were reported in [38,39]. Thus, the following assessment will be focused on the current bridge design specification (JTG D60−2015). As for single-truck load effects (see subgraphs of Figure 6), the load effects calculated due to the current design truck load (55 t) are the most significant, followed by the 49 t six-axle truck, the 43 t five-axle truck, and the 42.5 t five-axle truck. This trend can be expected because the load effect is entirely dependent on the magnitude of gross vehicle mass. Notably, the design load effects for some Grade-II highway bridges can be exceeded by those induced by a single design truck, as marked by square symbols.

### 5.2. ATP-Induced Load Effects on Simply Supported Bridges

**Figure 7:**
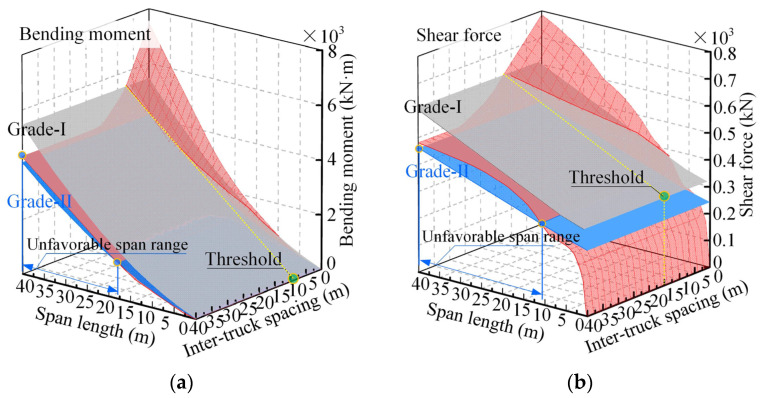
Load effects of simply supported bridges due to two-truck (55 t) ATP loads: (**a**) bending moment; (**b**) shear force.

Figure 7 plots the bending moments and shear forces of different simply supported bridges due to the two-truck (55 t) ATP loads spaced at different inter-truck spacings. The load effects were compared with those due to the design lane loads in the current bridge design specification (JTG D60−2015). As can be seen from Figure 7, the amplitudes of the ATP-induced load effects increase with the increase in bridge span length and the decrease in the inter-truck spacing. As the inter-truck spacing of the ATP is reduced to a certain limit, the load effects of the simply supported bridges due to the current design lane loads can be exceeded by those due to the ATP loads. In reality, the large limits are vulnerable. Under the same loading cases, the inter-truck spacing limits identified based on the shear forces are larger than those identified based on the bending moments, especially for Grade-II highway bridges. Therefore, shear force was regarded as the controlling internal force for assessing the impacts of ATP on simply supported bridges.

For Grade-I highway bridges, since the inter-truck spacing limits increase with the increase in bridge span lengths, the limit obtained from a 40 m simply supported bridge can reflect the minimum inter-truck spacing required to be maintained for the ATPs, which is called “inter-truck spacing threshold” in the following text. It can serve as a uniform guideline to control the minimum inter-truck spacing of the ATP on simply supported bridges. However, the obtained inter-truck spacing threshold only works for Grade-I highway bridges, as the load effects induced by the ATP on some Grade-II highway bridges can always be higher than those due to the design lane loads (2015 version). For these Grade-II highway bridges, the span range is called “unfavorable span range” hereafter and is illustrated in Figure 7. As can be seen from Figure 7, under the action of the two-truck (55 t) ATP loads, the unfavorable span range of simply supported bridges identified based on the bending moment is about 17–40 m, and that based on the shear force is about 11–40 m.

#### 5.2.1. Analysis of Unfavorable Span Range

**Figure 8:**
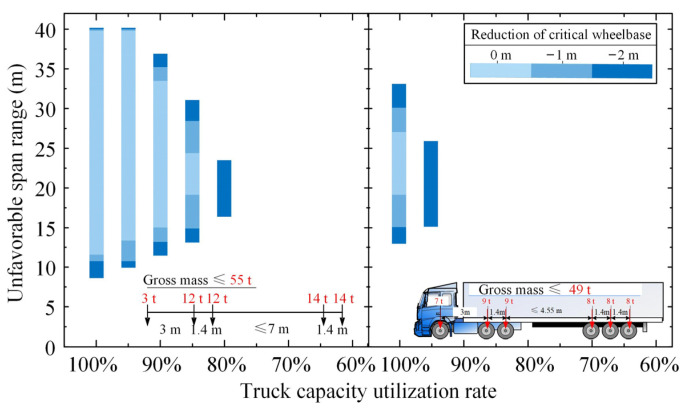
Unfavorable span ranges of Grade-II simply supported bridges under the action of ATP loads consisting of design truck loads and six-axle truck loads.

As mentioned previously, the unfavorable span ranges only exist in some Grade-II simply supported bridges. Figure 8 plots the unfavorable span ranges of Grade-II simply supported bridges under the action of ATP with different truck capacity utilization rates and different reductions in the critical wheelbase. In addition, only the unfavorable span ranges of the bridges under the action of ATP composed of 55 t design trucks and 49 t six-axle trucks are illustrated, because the two types of five-axle trucks even in a full load state will not lead to the unfavorable span ranges.

As can be seen in Figure 8, the unfavorable span ranges reduce with the decrease in truck capacity utilization rate but increase with the reduction in the critical wheelbase. Specifically, the unfavorable span ranges of simply supported bridges can be entirely eliminated when the truck capacity utilization rate decreases to 80% for the ATP with 55 t design trucks and 95% for the ATP with 49 t six-axle trucks. This indicates that overloaded trucks (heavier than 49 t) should be prohibited in ATP applications, and the six-axle semi-trailer trucks should be subjected to a new upper mass limit of 46 t (corresponding to a capacity utilization rate of 95%). In any case, the five-axle semi-trailer trucks with gross vehicle masses less than 43 t (equaling the corresponding legal limit) can be first applied, since they will not cause the phenomena of unfavorable span ranges.

#### 5.2.2. Analysis of Inter-Truck Spacing Threshold

In addition to avoiding the unfavorable bridge span ranges by controlling the mass of platooning trucks, the inter-truck spacing of the ATP should also be maintained in a reasonable range to balance the fuel efficiency and the collision risk of the ATP. Similar to the unfavorable bridge span range, the inter-truck spacing threshold can also be affected by the reduction in the critical wheelbase and the truck capacity utilization rate.

**Figure 9:**
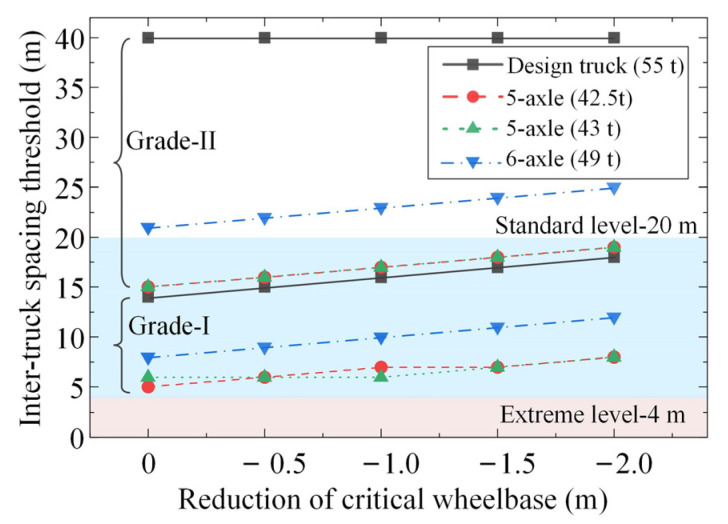
Inter-truck spacing thresholds for ATP crossing simply supported bridges with different reductions in the critical wheelbase (identified based on the shear force under the action of two-truck ATP with full loads).

Figure 9 plots the inter-truck spacing thresholds for ATP crossing simply supported bridges with different reductions in the critical wheelbase. It can be seen from Figure 9 that the inter-truck spacing thresholds increase linearly with the reduction in the critical wheelbase because the reduction in the critical wheelbase causes a more concentrated distribution of the ATP loads. This emphasizes the necessity of prohibiting an over-short critical wheelbase for platooning trucks.

**Figure 10:**
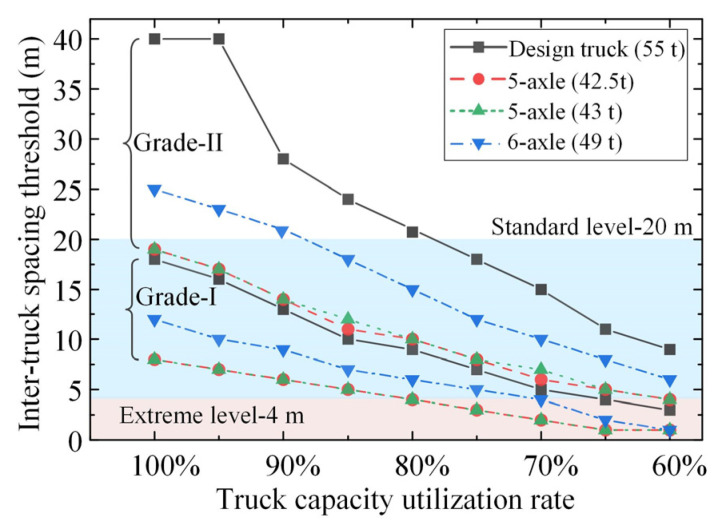
Inter-truck spacing thresholds for ATP crossing simply supported bridges with different truck capacity utilization rates (identified based on the shear force under two-truck ATP loads with a reduction of 2 m in the critical wheelbase).

Figure 10 plots the inter-truck spacing thresholds for ATP crossing simply supported bridges with different truck capacity utilization rates. Since the inter-truck spacing thresholds are not further affected by the ATPs composed of more than two trucks due to the limit of bridge span length, the analyses were based on the shear forces of the simply supported bridges under the action of two-truck ATP loads, and a reduction of 2 m in the critical wheelbase was considered. It can be seen from Figure 10 that the inter-truck spacing thresholds gradually decrease with the decreasing truck capacity utilization rate, indicating that all highway bridges can accommodate ATP loads as the capacity utilization rate of each platooning truck, which reflects the truck weight, is reduced to a certain value. Under the same ATP loads, the inter-truck spacing thresholds identified for Grade-II highway bridges are higher than those identified for Grade-I highway bridges, especially for the ATP loads consisting of 55 t and 49 t truck loads. Specifically, inter-truck spacings larger than 20 m can be adopted for both types of five-axle trucks with gross masses below 43 t. As for the six-axle truck, if this type of truck has to be applied in the ATP with an inter-truck spacing as small as 20 m, it is only suggested to be implemented on Grade-I simply supported highway bridges unless its gross mass is reduced to below 44.1 t (corresponding to a capacity utilization rate of 90%). If this type of truck under full load has to be applied on Grade-II simply supported bridges, the minimum inter-truck spacing of its corresponding ATP should be strictly controlled to be larger than 25 m.

#### 5.2.3. Suggestions on ATP Crossing Simply Supported Bridges

Avoiding unfavorable span ranges by limiting truck mass and keeping the inter-truck spacing threshold within a reasonable range are of equal importance for making simply supported highway bridges accommodate ATP loads. Based on the results in Figure 8 and Figure 10, it was found that both of those targets can be achieved by limiting the gross truck mass (truck capacity utilization rate). For this reason, the capacity utilization rates (mass limits) of trucks that can avoid the unfavorable span ranges and maintain a standard inter-truck spacing of 20 m are listed in Table 2. For Grade-I simply supported bridges, all legal trucks can be applied to the ATP scenarios, and the minimum inter-truck spacing for the platooning trucks with a full load can be as short as 13 m for the six-axle trucks and 8 m for the two types of five-axle trucks. As for Grade-II simply supported bridges, with an inter-truck spacing no less than 20 m, the five-axle trucks are recommended to be firstly applied, while the six-axle trucks with an upper mass limit of 44 t can also be adopted.

### 5.3. ATP-Induced Load Effects on Continuous Bridges

**Figure 11:**
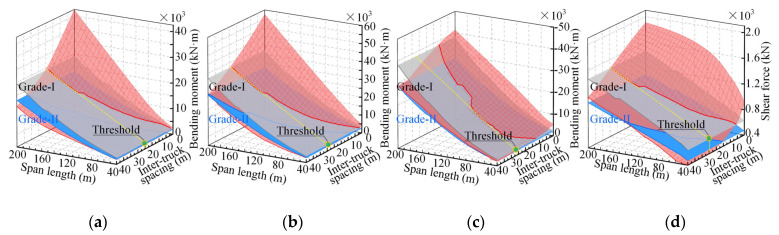
Load effects of continuous bridges due to four-truck (55 t) ATP loads: (**a**) bending moment at middle section of the side span; (**b**) bending moment at middle section of the main span; (**c**) negative bending moment at the interior support; (**d**) shear forces at the interior support.

In addition to simply supported bridges, continuous bridges were also considered. Figure 11 provides 3D surfaces for the load effects of ATP on three-span continuous bridges. The side-to-main span ratio of these continuous bridges was 0.7. Only the load effects (four controlling internal forces) due to four-truck (55 t) ATP loads were presented for illustration. It can be seen from Figure 11 that all four controlling internal forces due to the design lane loads (2015 version) are significantly exceeded by those due to the ATP loads and that adjusting the inter-truck spacing may be a good choice to reduce the load effects.

#### 5.3.1. Analysis of Inter-Truck Spacing Thresholds

**Figure 12:**
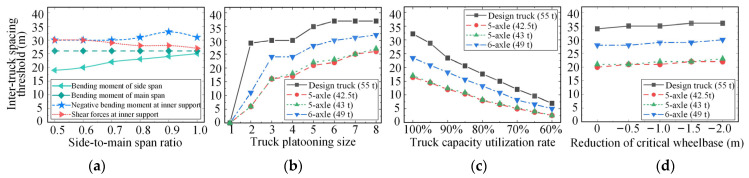
Parametric analysis of inter-truck spacing threshold: (**a**) effect of side-to-main span ratio; (**b**) effect of truck platooning size; (**c**) effect of truck capacity utilization rate; (**d**) effect of reduction in the critical wheelbase.

Figure 12 shows the effect of four parameters (i.e., the side-to-main span ratio of bridges, the truck platooning size, the truck capacity utilization rate, and the reduction in the critical wheelbase) on the inter-truck spacing thresholds. From Figure 12a, it can be seen that the inter-truck spacing thresholds identified based on the negative bending moments at the interior support are greater than those identified based on other internal forces, especially when the side-to-main span ratio of the continuous bridge is larger than 0.7. Combined with the results in Figure 11c, it can be inferred that the negative bending moment over the interior support is the most critical controlling internal force when assessing the load effect of ATP on continuous bridges. Similar findings were also reported in [22,40].

Figure 12b shows that the inter-truck spacing thresholds increase significantly with the increase in truck platooning size and then stabilize at certain values when the truck platooning size reaches seven. This may be due to the fact that the main span length of the continuous highway bridges is relatively long, and the ATP loads imposed on the same or adjacent spans can contribute to larger negative bending moments at the interior support. Thus, continuous bridges are more sensitive to the platooning size of ATP than simply supported bridges. As can be seen from Figure 12c,d, the effects of truck capacity utilization rate and reduction in the critical wheelbase on the inter-truck spacing threshold identified for continuous bridges are similar to those for simply supported bridges. The inter-truck spacing thresholds decrease significantly with the decrease in the truck capacity utilization rate but increase linearly with the reduction in the critical wheelbase. Both of these factors, together with the platooning size, should be considered in adjusting the inter-truck spacing of ATP.

#### 5.3.2. Suggestions on ATP Crossing Continuous Bridges

**Figure 13:**
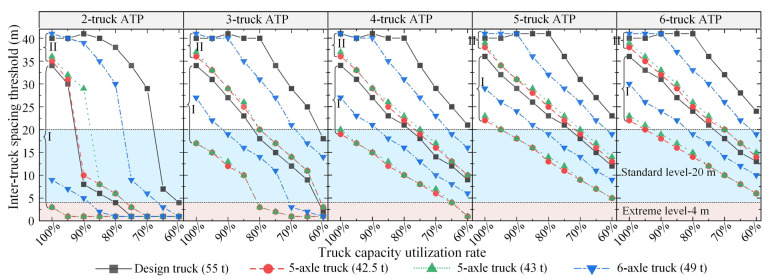
Inter-truck spacing thresholds for ATP crossing continuous bridges with different truck capacity utilization rates and different platooning sizes.

Figure 13 plots the inter-truck spacing thresholds, identified based on the negative bending moments at the interior support, of ATP crossing continuous bridges with different platooning sizes (2–6). The side-to-main span ratio of the continuous bridges was taken as 0.7, and a reduction of 2 m in the critical wheelbase was considered. As can be seen from Figure 13, the inter-truck spacing thresholds for each considered ATP decline with the decrease in truck capacity utilization rate, and the inter-truck spacing thresholds for different considered ATPs vary significantly at the same capacity utilization rate. Specifically, a two-truck ATP with the standard inter-truck spacing (20 m), regardless of the truck type, may be allowed to travel across Grade-I continuous bridges. However, the Grade-I continuous bridges may be inadequate to sustain the load of a longer ATP formation. For continuous bridges to be able to accommodate the loads of ATP with the standard inter-truck spacing of 20 m, additional mass limits are suggested to be imposed on the five-axle and six-axle trucks when the number of platooning trucks is greater than four and two, respectively. As for Grade-II continuous bridges, both the inter-truck spacing and the truck platooning size of all four types of trucks should be considered carefully when deciding the final capacity utilization rate for each platooning truck.

To facilitate the application of the ATP, Table 3 summarizes the suggestions on the truck platooning size, inter-truck spacing, truck type, and corresponding upper mass limits to protect the Grade-I and Grade-II highway bridges from adverse impacts induced by the ATP loads.

### 5.4. Suggested General Upper Mass Limits

**Figure 14:**
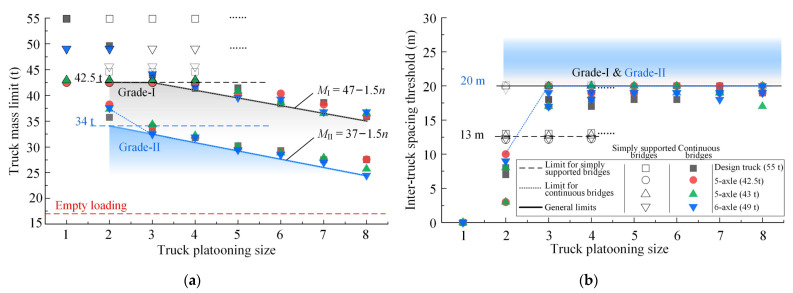
Identification of the general upper mass limits for platooning trucks crossing highway bridges with the standard inter-truck spacing of 20 m. (**a**) the general upper mass limits for platooning trucks with a standard inter-truck spacing of 20 m; (**b**) the corresponding limits on the inter-truck spacings.

Proposing general suggestions on ATP crossing both simply supported and continuous highway bridges is of great significance in practice. For this purpose, the suggested upper mass limits of three types of typical semi-trailer trucks (excluding the design truck) traveling across these two types of bridges are illustrated in Figure 14. For ATPs with the same platooning size, if the maximum inter-truck spacing is no less than 20 m, the minimum of the suggested upper mass limits for different types of trucks can serve as a general limit of masses for road trucks, which is called “general upper mass limit” in the following text. Based on this, all general upper mass limits for platooning trucks crossing different grades of highway bridges with different platooning sizes were identified. The relationships between the general upper mass limits of platooning trucks and the corresponding truck platooning sizes can be expressed by Equation (2):

(2) $$\left\{\begin{array}{c} M_{I}=\left|\begin{array}{l} \;42.5\;,\;\;\left(n\leq 3\right)\\ \;47-1.5n,\;\;\left(n\geq 3\right) \end{array}\right.\\ M_{\mathrm{II}}=\;\;\;37-1.5n,\;\;\left(n\geq 2\right) \end{array}\right.$$

in which *M* is the general upper mass limit for platooning trucks and *n* denotes the truck platooning size.

According to Equation (2), if the truck platooning size does not exceed three, the truck mass limit can be uniformly taken as 42.5 t, which is equal to the current legal upper mass limit for five-axle trucks [34]. Therefore, it is recommended to limit the truck platooning size to three trucks. The general upper mass limits suggested for other scenarios of ATP crossing highway bridges can be taken according to Equation (2). For the same ATP configurations, the general mass limit suggested for Grade-II bridges is lower by 10 t than that suggested for Grade-I highway bridges.

With the advancement of the V2V communication technology, the inter-truck spacings (following gaps) of the ATP can be shortened and organized more flexibly, and thus a more comprehensive regulation of the upper mass limits for platooning trucks will be needed. Table 4 lists the general upper mass limits identified under different combinations of the truck platooning size and the following gap (=inter-truck spacing − 5 m). As can be seen from Table 4, a shorter following gap and a larger truck platooning size generally cause a lower general upper mass limit. For ATPs arranged with the standard following gap (15 m), if the truck platooning size is not greater than three trucks, the current legal upper mass limit (42.5 t) can still be applied. For the ATPs composed of more than three trucks, the following gaps are suggested accordingly to apply the current legal upper mass limit (42.5 t). It should be noted that a maximum truck platooning size of three is also suggested in the Eurocode (2019) [41] and is legally permitted by the State of Pennsylvania in the U.S. [42].

## 6. Loading Strategy for ATP in Highway Cargo Transport

**Figure 15:**
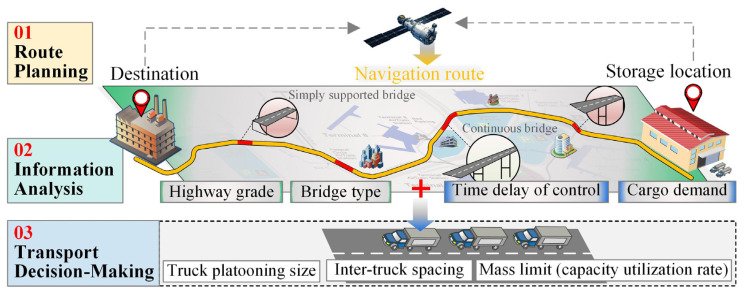
Loading strategy for ATP transporting cargo on a highway.

A cargo loading strategy for ATP transport is proposed based on the above-obtained results in this section, as illustrated in Figure 15. The cargo loading strategy includes three steps:

1. Transportation route planning:
  - Input the destination to the navigation system and find the possible routes;
  - Determine the best route for platooning trucks from an economical perspective.
2. Information collection and analysis:
  - Obtain detailed bridge information (including the specific bridge type and the highway grade) for the selected route;
  - Acquire the cargo volume from the supplier, determine the exact number of trucks required for transportation, and measure the time delay of the vehicular communication system to determine the stable inter-truck spacing that should be maintained.
3. Transport decision-making:
  - Decide the truck platooning size and the inter-truck spacing for ATP in cargo transport;
  - Based on the collected information above and the findings of this study, determine the capacity utilization rate for each platooning truck.

## 7. Conclusions

To investigate the potential adverse impact of the newly developed automated truck platooning (ATP) technique, in this study, the responses of Chinese highway bridges under the action of ATP loads were studied by comparing the load effects due to ATP loads with those due to the current design lane loads (2015 version). The minimum inter-truck spacing and the suggested upper mass limits for platooning trucks crossing highway bridges were identified. The main conclusions drawn are as follows:

(1) Without a proper arrangement, the load effects due to the ATP loads may exceed those due to the design lane loads. The ATP-induced load effects increase with the increase in the truck platooning size and the reduction in the inter-truck spacing. To ensure the safety of the highway bridges, more caution needs to be paid to ATP arrangements, including selecting the truck type, controlling the inter-truck spacing, limiting the number of platooning trucks, and deciding the truck capacity utilization rate.
(2) Based on the current Chinese legal limits of masses and dimensions for trucks, ATP is recommended to be first applied to Grade-I highway bridges. In this case, the five-axle trucks can be adopted with priority, and the suggested platoon form is three trucks operating with an inter-truck spacing larger than 20 m. For ATPs consisting of more trucks or spaced at shorter inter-truck spacings, additional upper mass limits should be enforced, especially when they are applied to Grade-II highway bridges.
(3) A general cargo loading strategy for ATP transportation is proposed in this study. Based on the bridge information and the cargo transport demands (including the cargo volume, truck type, and the time delay of vehicular communication), the ATP transport can be well managed in a form that does not cause adverse impacts on existing highway bridges.

In this study, the impact of the ATP loads on existing highway bridges was preliminarily assessed. With the rapid growth of the market demand for ATP transportation, more complicated scenarios of ATP loading on highway bridges will occur. To ensure the safety of both bridge structures and truck operations, more efforts need to be made to investigate the ATP–bridge coupled vibration system in future work.

## Figures and Tables

**Table 1:** Parameter setting for assessing load effects of highway bridges under ATP loads.

Objective	Parameter	Range
Truck	Truck type	① Design truck (≤55.0 t) ② 5-axle truck (≤42.5 t) ③ 5-axle truck (≤43.0 t) ④ 6-axle truck (≤49.0 t)
	Reduction in critical wheelbase	0–2 m
	Truck capacity utilization rate	60–100%
	Number of platooning trucks	1–8
	Inter-truck spacing	0–40 m
	Standard inter-truck spacing	20 m
	Extremum inter-truck spacing	4 m
Simply supported bridge	Span length	0–40 m
	Controlling internal force	Bending moment at the mid-span Shear force at the side support
Continuous bridge	Main span length	40–200 m
	Side-to-main span ratio	0.5–1.0 [37]
	Controlling internal force	Bending moment at the middle section of the side span Bending moment at the middle section of the main span Shear force at the interior support Negative bending moment at the interior support

**Table 2:** Identified mass limits for platooning trucks crossing simply supported bridges.

Highway Bridges	Truck Type	Target		Suggestions
		Avoid Unfavorable Span Ranges	Accommodate Standard Inter-Truck Spacing Level (20 m)	
Grade-I	①	100% (55.0 t)	100% (55.0 t)	keep inter-truck spacing ≥ 13 m without additional mass limits
	②	100% (42.5 t)	100% (42.5 t)	
	③	100% (43.0 t)	100% (43.0 t)	
	④	100% (49.0 t)	100% (49.0 t)	
Grade-II	①	80% (44.0 t)	80% (44.0 t)	keep inter-truck spacing ≥ 20 m keep the mass of six-axle truck ≤ 44 t
	②	100% (42.5 t)	100% (42.5 t)	
	③	100% (43.0 t)	100% (43.0 t)	
	④	95% (46.5 t)	90% (44.0 t)	

**Table 3:** Suggested upper mass limits (t) for platooning trucks crossing continuous bridges.

Truck Platooning Size	Truck Type	Grade-I Highway Bridge		Grade-II Highway Bridge	
		Inter-Truck Spacing = 20 m	Inter-Truck Spacing = 4 m	Inter-Truck Spacing = 20 m	Inter-Truck Spacing = 4 m
2	①	49.5	41.3	35.8	33.0
	②	42.5 *	42.5	38.3	31.8
	③	43.0 *	43.0	38.7	32.2
	④	49.0 *	41.6	36.8	31.8
3	①	44.0	33.0	33.0	22.0
	②	42.5 *	34.0	34.0	25.5
	③	43.0 *	34.4	34.4	25.8
	④	44.1	34.0	31.9	22.1
4	①	41.3	27.5	30.2	22.0
	②	42.5 *	25.5	31.9	19.6
	③	43.0 *	25.8	32.2	19.3
	④	41.7	24.5	31.9	19.1
5	①	38.5	24.7	30.2	19.2
	②	40.4	21.2	29.7	17.0
	③	40.9	21.5	30.1	17.2
	④	39.2	21.0	29.4	17.0
6	①	38.5	22.0	30.2	19.2
	②	40.4	23.4	29.7	17.3
	③	38.7	23.6	30.1	17.2
	④	39.2	22.1	29.4	17.1
7	①	38.5	19.2	27.5	13.7
	②	38.2	21.2	27.6	17.0
	③	36.8	21.5	27.9	17.2
	④	36.7	19.6	26.9	14.7
8	①	35.7	19.2	27.5	13.7
	②	36.1	19.1	25.5	17.0
	③	36.5	19.3	25.8	17.2
	④	36.1	19.0	25.0	14.7

* denotes a value equals to the corresponding legal limit.

**Table 4:** Suggested upper mass limits (t) for platooning trucks under different scenarios.

Highway Bridge	Following Gap ^1^	Truck Platooning Size						
		2-Truck	3-Truck	4-Truck	5-Truck	6-Truck	7-Truck	8-Truck
Grade-I	0	25.0	25.0	24.0	21.0	22.0	19.0	19.0
	5 m	36.0	35.0	34.0	29.0	29.0	25.0	24.0
	10 m	42.5 *	39.0	36.0	34.0	34.0	31.0	31.0
	15 m	42.5 *	42.5 *	41.0	39.5	38.0	36.5	35.0
	20 m	42.5 *	42.5 *	42.5 *	42.5 *	42.5 *	40.0	40.0
	25 m	42.5 *	42.5 *	42.5 *	42.5 *	42.5 *	42.5 *	42.5 *
Grade-II	0	17.0	17.0	17.0	17.0	17.0	14.0	14.0
	5 m	25.5	25.5	24.0	22.5	21.0	19.5	19.0
	10 m	31.0	29.0	26.0	25.0	25.0	24.0	22.0
	15 m	34.0	32.5	31.0	29.5	28.0	26.5	25.0
	20 m	34.0	34.0	34.0	31.0	31.0	29.0	29.0
	25 m	34.0	34.0	34.0	34.0	34.0	34.0	31.0

^1^ following gap = inter-truck spacing − 5 m (the total length of the front and rear overhang of a truck); * denotes a value equals to the corresponding legal limit.

## Data Availability

Not applicable.
